# Eye Tracking Insights Into Movement Preparation and Execution Under Nonstandard Visual Movement Feedback

**DOI:** 10.1111/psyp.70373

**Published:** 2026-08-02

**Authors:** Felix Quirmbach, Jens R. Helmert, Sebastian Pannasch, Annika Dix, Jakub Limanowski

**Affiliations:** ^1^ Faculty of Psychology TUD Dresden University of Technology Dresden Germany; ^2^ Centre for Tactile Internet With Human‐In‐The‐Loop (CeTI) TUD Dresden University of Technology Dresden Germany; ^3^ Chair of Automobile Engineering TUD Dresden University of Technology Dresden Germany; ^4^ Institute of Psychology University of Greifswald Greifswald Germany

**Keywords:** action, eye tracking, movement preparation, virtual reality, visuomotor mapping

## Abstract

For eye‐hand coordination, predictions of sensory movement consequences may already be issued, and adjusted, during action preparation. In this pre‐registered study, we combined a delayed‐movement paradigm with a virtual reality‐based eye‐hand tracking task to investigate the oculomotor correlates of planning and executing coordinated eye‐hand movements under standard versus nonstandard visual hand movement feedback. We measured pupil dilation and gaze‐hand tracking during action preparation and subsequent task execution, where visual movement feedback violated or matched cued expectations: Participants prepared and, after a delay period, executed hand movements. Their movements were reflected by congruent or incongruent (inverted) movements of a glove‐controlled virtual hand model, which they had to follow with their gaze. In the preceding delay period, visual cues could specify the to‐be‐executed movement (or leave it unspecified), and the visuomotor mapping (congruent or incongruent, 75% cue validity). We found that during the delay, pupil diameter increased more strongly when the movement was pre‐cued (compared to left unspecified), and when nonstandard compared with standard visual movement feedback was expected. During execution, gaze‐hand tracking performance decreased under nonstandard mappings, but significantly less so when the to‐be‐executed movement was pre‐cued. Expectation violation trials produced a significantly stronger pupil dilation, particularly when congruent (standard) visuomotor expectations were violated, but also when incongruent mappings were cued but congruent ones observed. Furthermore, expectation violation impaired tracking performance; again, more strongly when congruent visuomotor expectations were violated. Our results indicate that oculomotor responses can reflect processes related to motor planning and flexible forward prediction of sensory action consequences ahead of execution, that is, increased mental effort and expectations of sensory conflict. Moreover, our results suggest that specific expectations about visuomotor (in)congruency affect eye‐hand coordination and pupillary responses during subsequent execution of the planned action.

## Impact Statement

Our study provides novel insights into the planning and execution of coordinated eye‐hand movements by showing that oculomotor responses reflect predictive processes related to motor planning under expected standard versus nonstandard visual movement feedback, and relating these predictions to eye‐hand coordination during subsequent execution of the planned action.

## 1 Introduction

Performing everyday manual actions like grasping an object or catching a ball requires the coordinated control of hand and eye movements. This often involves tracking the hand's movements with one's gaze. Thus, besides coordinating the motor commands issued to the different effectors, it requires an accurate prediction of the sensory data generated by the effectors' movements. In other words, it relies on an adequate prediction of where the hand will be seen and what the hand movement will look like. The prediction of sensory action consequences is thought to be enabled by internal forward models in the brain (Miall and Wolpert 1996; Shadmehr and Krakauer 2008; Wolpert and Kawato 1998). These forward models are based on life‐long learning, yet they are also surprisingly flexible. For instance, participants can quickly adapt to novel sensorimotor mappings, such as when learning to move under nonstandard (e.g., rotated or delayed) visual movement feedback (e.g., Bock 2013; Cunningham 1989; Limanowski and Friston 2020). This learning process happens through updating forward models by sensory prediction errors; that is, unpredicted sensory action consequences (Grafton et al. 2008; Shadmehr et al. 2010; Synofzik et al. 2006; Tseng et al. 2007). Notably, the concept of forward sensory predictions extends to action preparation; that is, generating sensory predictions during motor planning—even in the absence of actual movement and related sensory feedback (Kilteni et al. 2018; Kuang et al. 2016; Sawtell 2017; van Kemenade et al. 2016). Recent brain imaging and electrophysiological work using delayed‐movement paradigms has shown that the preparation of (delayed) actions under nonstandard visual movement feedback is associated with increased activity in brain regions thought to implement forward models; that is, the cerebellum and posterior parietal cortex (Kuang et al. 2016; Pilacinski et al. 2018; Quirmbach and Limanowski 2024). This implies that the brain's forward models can anticipate novel (“unpredicted”) sensory action consequences, and adjust their respective sensory predictions ahead of execution.

Besides central nervous activity, oculomotor behavior can reveal much about the mental processes associated with sensory prediction, sensorimotor integration, and their role for eye‐hand coordination. Particularly promising is pupil dilation, as a distinct readout of locus coeruleus‐noradrenergic responses related to executive control (Alnæs et al. 2014; Grujic et al. 2024). Thus, pupil dilation is considered a reliable marker of the (task‐relevant) uncertainty of internal states (Becker et al. 2024; Fan et al. 2023; Preuschoff et al. 2011; Satterthwaite et al. 2007), and of mental effort (Aston‐Jones and Cohen 2005; Joshi and Gold 2020; Shenhav et al. 2017), also in sensorimotor tasks (Hosseini et al. 2017; cf. Zénon et al. 2014). Pupil dilation increases when responses are pre‐cued (Adam et al. 2014; Moresi et al. 2008), and pupil diameter can reflect the anticipated difficulty or the expected reward of a task (Dix and Li 2020; Irons et al. 2017; Spliethoff et al. 2022). Furthermore, expectation violation reliably evokes pupil dilation (Alamia et al. 2019; Bianco et al. 2020; Braem et al. 2015; Kloosterman et al. 2015; Reisenzein et al. 2019; Yokoi and Weiler 2022), and error‐based motor learning is reflected by changes in trial‐by‐trial pupil dilation (Yokoi and Weiler 2022). This suggests a possible link of pupillary responses to the updating of sensorimotor associations and the sensory predictions of internal (forward) models. Specifically, pupil size could indicate the mental effort and the (un)certainty associated with generating (nonstandard) sensory forward predictions.

Pupil size has been measured to assess anticipatory mental effort during saccade preparation in a delayed movement task, but without concurrent hand movements (Hutchison et al. 2020; Jainta et al. 2011; Unsworth et al. 2023; Wang et al. 2015, 2016). Several eye tracking studies have used anti‐saccade designs to decouple hand and eye movement goals; that is, requiring the execution of eye and hand movements to different (versus the same) targets or directions. These studies have yielded important insights into the on‐line coordination of hand and eye movements under various conditions, including saccadic versus smooth pursuit, or manipulated visual movement feedback (e.g., Chen et al. 2016b; Gorbet and Sergio 2009; Vercher et al. 1995; Yeomans, Yan, et al. 2021). However, these studies focused on on‐line control; that is, execution without prior planning or preparation. Thus, to our knowledge, no one has yet investigated oculomotor behavior during the preparation (and subsequent execution) of manual actions with nonstandard visual hand movement feedback.

**FIGURE 1:**
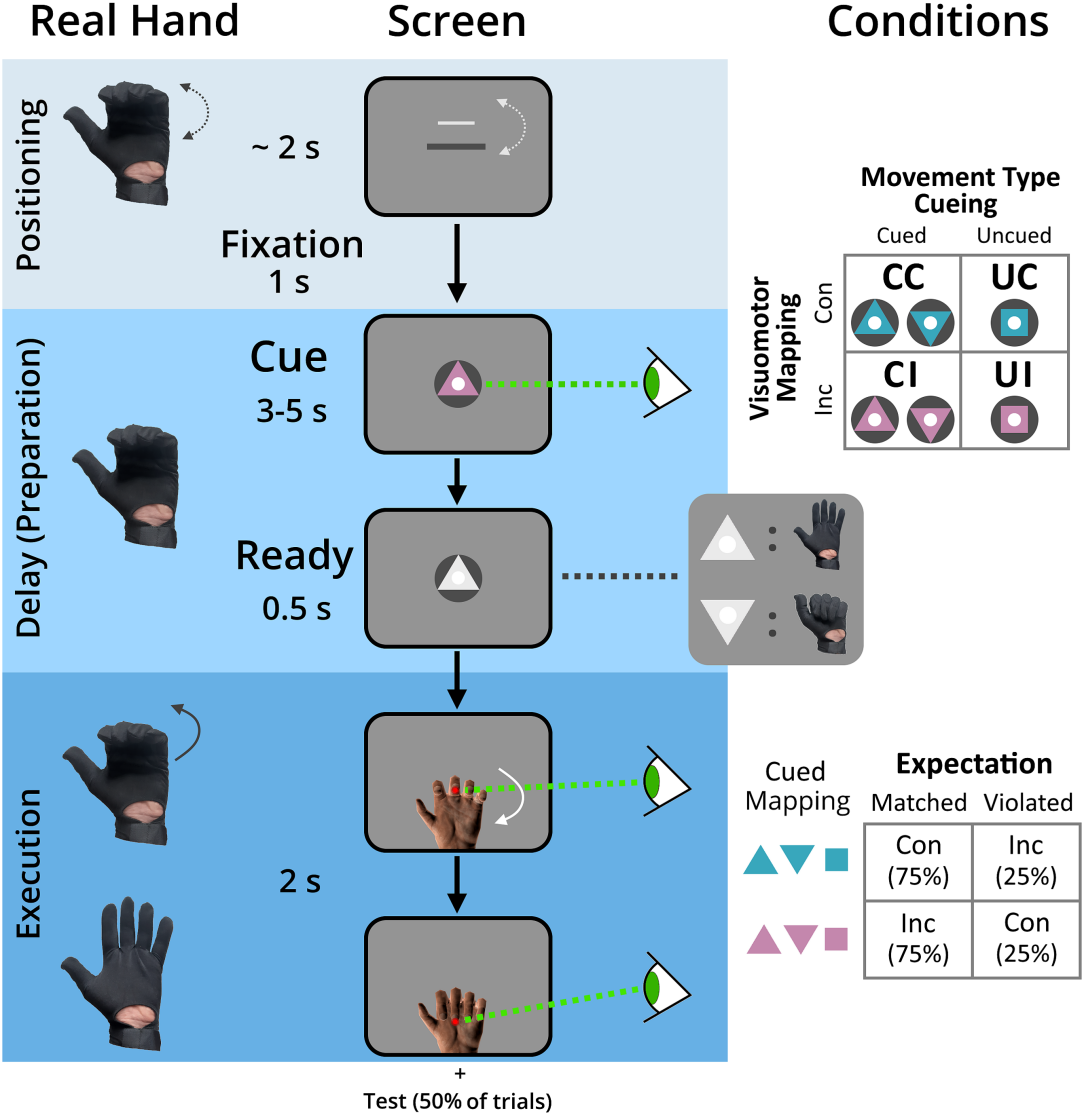
Task design. Schematic of an exemplary trial. The participants' unseen real hand movements were measured via data glove (left) and fed to a virtual hand model presented on screen (middle); with visual cues (right) predicting different aspects of the upcoming, to‐be‐executed movement. To begin a trial, participants moved their hand to a neutral, half‐opened starting position, by aligning a moving bar with a target (see Methods). After a 1 s fixation‐only period, a visual cue was then presented for 3–5 s. The cue color (cyan or pink) indicated the visuomotor mapping during subsequent movement execution: In 50% of trials, the virtual hand movement would reflect the movements of the real hand (congruent condition, here: Cyan), in the other 50% its movement was inverted (incongruent condition, here: Pink). The cue shape could predict the to‐be‐executed hand movement: Either opening (upward pointing triangle, 25%) or closing it (downward triangle, 25%). In the remaining 50% of trials, a square cue shape left the to‐be‐executed movement unspecified. After the delay period, a brief (0.5 s) ‘Ready’ signal (white triangle) appeared, indicating that movement execution would follow immediately and instructed subjects to either open (triangle pointing up) or close (triangle pointing down) their real hand—in cued trials, this would confirm the previous movement type, while signaling it for the first time in uncued trials. Subjects then had to execute the instructed hand movement during a 2 s time period following the appearance of the virtual hand. During hand movement execution, participants had to follow a red dot at the middle finger tip of the virtual hand with their gaze. As cued during delay, the virtual hand moved either congruently or incongruently (i.e., inverted) to the real hand. Importantly, the mapping cue was only valid in 75% of trials. In the remaining 25% of trials, the virtual hand moved opposite to what was cued (e.g., congruently, although an incongruent mapping had been cued), to violate cued visuomotor expectations. Overall, this resulted in a full factorial, within‐subject design with factors *Movement type cueing* (Cued, Uncued) and *Visuomotor mapping* (Congruent, Incongruent), and additional nested factors specifying *Movement type* (Open, Close) and *Expectation violation* (Matched, Violated). Stimuli and virtual display not to scale.

Therefore, in this pre‐registered study (https://doi.org/10.17605/OSF.IO/6KM9Y), we built upon the above works by investigating the oculomotor correlates of preparing and executing manual movements under nonstandard visual hand movement feedback, under varying (cued) certainty of the underlying movement plans. For this, we employed a novel eye‐hand tracking task (Figure 1): Participants controlled a photorealistic virtual hand via a data glove worn on their unseen hand, while tracking the motion of the virtual hand with their gaze. Crucially, we embedded this task in a delayed‐movement paradigm; that is, participants prepared the to‐be‐executed hand movements and executed them after a delay. During the delay (preparation period), visual cues could specify the to‐be‐executed movement and the expected visuomotor mapping. Specifically, the cue shape could indicate the preparation of an open or a close hand movement (or leave it unspecified), while the cue color indicated whether, in the upcoming execution period, the virtual hand would display the participant's actual executed hand movement (congruent mapping) or an inverted movement (incongruent mapping). Importantly, the virtual hand movements were always controlled by the participant; that is, in the incongruent trials, the virtual hand received the same glove sensor values, only inverted. Thus, for efficient gaze‐hand tracking, participants needed to generate the appropriate visuomotor predictions; that is, movement plans and predictions of visual hand movement feedback. In the incongruent conditions, the predictions of how the executed hand movement will look like differed from the standard (life‐long learned) association and, consequently, required updating for subsequent gaze‐hand tracking. Finally, we added “expectation violation” trials, in which the virtual hand moved contrary to the cued visuomotor mapping, and thus, the visual movement feedback violated the participants' expectations. During the task, we recorded hand and gaze movements, as well as pupil diameter. We hypothesized that these measures would indicate the mental effort involved in anticipating conflicting (nonstandard) visual movement feedback and updating visuomotor expectations during planning; and how these factors influenced gaze‐hand coordination during subsequent task execution. Specifically, we had six key hypotheses:

During the delay phase (movement preparation), we expected greater pupil dilation to be evoked by cueing incongruent > congruent visual movement feedback (main effect, *Hypothesis H1*), reflecting the anticipation of visual hand movement feedback conflicting with life‐long visuomotor expectations, the potential updating of forward sensory predictions, and the mental effort associated with correspondingly increased cognitive control (cf. Shenhav et al. 2017; Yon et al. 2018). We also expected increased pupil dilation if the upcoming movement was cued > uncued (main effect, *Hypothesis H2*). This prediction followed from our previous study using a similar task design (Quirmbach and Limanowski 2024; cf. Rosenbaum 1980), where the movement data clearly suggested that participants prepared a specific movement if cued correspondingly, but abstained from (parallel) movement planning in the uncued condition. Furthermore, we expected an interaction effect; that is, the planning‐related (cued > uncued) pupillary dilation should be more pronounced in trials with incongruent compared to congruent visual feedback (*Hypothesis H3*).

During execution, we expected that a successful generation (and, for cued incongruent mappings, an updating) of visuomotor expectations during movement preparation would be associated with a “surprise response” to observing a visual movement contrary to that predicted by the cue (*Hypothesis H4*). This response should be reflected by larger pupil dilation and reduced gaze‐hand tracking accuracy in expectation violation trials (see above). Furthermore, we expected better task (oculomotor) performance (less gaze‐hand tracking error and faster initiation of hand and eye movements) for movements under congruent, compared to incongruent visuomotor mappings, as the former correspond to lifelong associations between motor commands and visual feedback (*Hypothesis H5*). Previous work has shown that eye tracking of a target controlled by one's hand movements worsens significantly when the target's movement is manipulated with respect to the actually executed movement (Chen et al. 2016b; Vercher et al. 1995; Yeomans, Yan, et al. 2021). Thus, despite potential anticipatory processes and updated expectations, we assumed a residual benefit of moving under standard versus nonstandard visual feedback. Finally, we expected improved task performance when the movement type was cued, compared to being left uncued in the preparation period, because a more specific movement plan could be generated during the respective delay (*Hypothesis H6*).

## 2 Materials and Methods

### 2.1 Participants

Forty healthy volunteers (23 female, mean age = 25.9 years, range 19–36, normal or corrected‐to‐normal vision) participated in the experiment after signing informed consent. Based on a recent study using a similar task design (Quirmbach and Limanowski 2024), we determined a minimum sample size of 36 participants to reach 95% power via with the software program G*power (Cohen's *f* = 0.25, alpha error: 0.05, 4 measurements per condition, correlation of 0.5 between measurements). One subject had to be excluded due to technical issues with their eye tracking measurements, leaving 39 participants' data sets for the group level analysis. As compensation for performing the study, participants received 20€, and up to 5€ depending on the percentage of catch trial questions answered correctly (1€ if they reached at least a 75% correct answer rate, 3€ for at least 85% correct and 5 € for at least 95% correct, see below). The experiment was approved by the ethics committee of the Technische Universität Dresden and conducted in accordance with this approval.

### 2.2 Pre‐Registration

This study was pre‐registered (osf.io/6km9y). Pre‐registration was done after data collection but before data analysis; that is, aside from confirming basic file readability, the content of data files was neither examined nor analyzed in any form prior to registration. Hypotheses, task design, and data analyses presented here follow the plan laid out by pre‐registration; all deviations from our original plan and exploratory analyses are clearly stated and explained in the article.

### 2.3 Experimental Design and Procedure

The study took place in a moderately lit room with constant, artificial lighting, keeping luminance conditions constant across time and sessions. Participants sat on a desk with their heads stabilized via a chin rest, their eyes distanced 93 cm from a screen (BenQ LCD XL2420T), resulting in a maximal viewing angle of 31.9° (horizontal) and 17.7° (vertical). On the screen, all visual stimuli, that is, instructions, visual cues, and the virtual hand model, were displayed with a spatial resolution of 1920 × 1080 pixels, at a refresh rate of 60 Hz. The virtual display was created with the Blender 3D graphics software package (https://www.blender.org/, Version 2.79) and its Python programming interface.

During the experiment, pupil size and gaze position were recorded monocularly (left eye) at 1000 Hz via a desktop‐mounted eye tracking system (EyeLink 1000 Plus, SR Research); a 9‐point calibration and validation procedure was performed at the start of each block. A data glove (5DT Data Glove 14 Ultra MRI, 60 Hz sampling rate, full‐speed USB 1.1 connection) placed on the participants' left hand measured each finger's flexion via seven sewn‐in optical sensors (two for each finger; one excluded due to technical issues). For each participant, data transformation from the glove was calibrated carefully prior to the actual experiment based on the possible range of flexion to ensure that movement of the real and virtual hand matched. After averaging across sensors to ensure smooth and coherent visual motion (cf. Limanowski and Friston 2020), its data were fed to a photo‐realistic virtual hand model shown on the screen, which participants were therefore able to control. By placing their gloved real hand under the table and restricting their head towards the screen, throughout the experiment, they received visual feedback only from the virtual hand. The experimenter continuously observed both the virtual display and the participant from a distance, ensuring task compliance.

Figure 1 shows an example trial, described in the following: At the beginning of each trial (hand positioning), participants had to bring their real, unseen hand to the neutral starting position (i.e., fingers half‐way closed). A small white bar presented on screen, whose vertical position was controlled via data glove by the real hand's posture, indicated to which degree the hand was opened or closed. To start the trial, this bar had to align with a fixed, central gray bar (see Figure 1); on average, this took 1.98 s (SD = 0.54 s). Once both bars were aligned, they were replaced by a central white fixation dot (0.310° × 0.310° of visual angle). Participants were instructed to keep their gaze on this dot throughout the entirety of the delay period.

After 1 s, a visual cue was presented centrally and remained visible throughout the delay period (3–5 s, jittered and pseudo‐randomized, evenly distributed across conditions). This cue provided two types of predictive information: In half of all trials, triangle‐shaped cues (width: 1.057°/height: 0.870° of visual angle) informed participants in advance which real hand movement they would have to perform, with an upward‐pointing triangle (64 trials, 25%) instructing them to open their hand, while a downward‐pointing triangle (64 trials, 25%) instructed them to close it. In the other 50% (128 trials), a square‐shaped cue (0.682° × 0.682° of visual angle) did not provide any information about the to‐be‐performed movement. Therefore, participants were able to generate specific predictions about the sensory feedback of the upcoming movement in only half of the trials. Cue shapes were designed such that their on‐screen area was identical and were presented at the same position. Simultaneously, cue color indicated if during execution the virtual hand would move congruently to the real hand (i.e., perform the same movement type), or incongruently (i.e., perform the opposite movement type). In the latter case recorded hand movement data were inverted, so that opening the real hand would close the virtual hand, and vice versa. This way, we ensured that even with incongruent visuomotor mappings, visual feedback was still completely predictable from motor commands, and participants would perceive the visual hand movements as caused by their own actions. Cue colors (cyan and pink) were selected to be near‐isoluminant; the color coding was balanced across participants.

Following the delay period, a centrally presented white triangle (width: 1.057°/height: 0.870° of visual angle, same as the cue) served as a ‘Ready’ signal for 500 ms, indicating the imminent execution phase. For trials with square‐shaped cues—when movement type was left ambiguous—the triangle would also indicate which movement to perform, with an upward‐pointing triangle instructing opening of the real hand, and a downward‐pointing triangle instructing them to close the hand. To ensure visual feedback of the entire movement, participants were instructed to only start their hand movement as soon as the ‘Ready’ signal and fixation dot disappeared, and the virtual hand model appeared instead. In the following execution period (2 s), participants had to perform the instructed hand movement while following a visual target (red dot) at the tip of the virtual hand's middle finger with their gaze as closely as possible. At the start of the execution phase, the virtual hand was displayed in a neutral starting position, so that the red dot was positioned exactly in the middle of the screen. The virtual display was furthermore designed to ensure that both movement types were equidistant when the hand movements were executed completely, that is, the gaze target would cover the same Euclidean distance on the screen. After finishing the hand movement, that is, completely opening or closing it, the hand was then to be kept in the final position until the end of the trial. Note that, while the dynamics of the virtual hand on the screen could, in principle, affect pupillary responses through luminance changes, in our comparisons the hand movement directions were counter‐balanced across task conditions. After the execution period ended, the virtual hand model disappeared, and a blank screen was presented for 2 s, marking an inter‐trial interval.

In the execution period, we included occasional expectation violation trials (25%, i.e., 64 trials, balanced across conditions). In those trials, the virtual hand displayed the opposite visuomotor mapping to that predicted by the cue color during the delay period; that is, moving congruently when an incongruent mapping was cued, and vice versa. The inclusion of these trials allowed us to examine the oculomotor effects of expectation violation, testing our *Hypothesis H4*.

Altogether, this study employed a full factorial, within‐subjects design with two factors: *Predicted visuomotor mapping* and *movement type cueing*, resulting in four main conditions: CC (Cued movement type, expecting congruent mapping), CI (Cued movement type, expecting incongruent movement), UC (Movement type not cued, expecting congruent mapping), and UI (Movement type not cued, expecting incongruent mapping). For analysis of both pupil size and tracking performance during movement execution, we also included the nested factors *movement type* (Hand opening, closing) and *visuomotor expectation violation* (Matched, Violated). Participants performed the task under all conditions, with equal amounts of trials with hand opening or closing, movement type being cued or uncued, and congruent or incongruent virtual hand behavior. Visuomotor cues were valid in 75% of trials (192 trials) and invalid in 25% (64 trials).

Finally, to ensure that participants were paying attention to the predictive cues (and therefore able to generate visuomotor predictions), we included “catch trials”, in which participants had to indicate if the mapping of the virtual hand matched the cue's predictions (cf. Yon et al. 2018). After all invalidly cued trials, and a matching number of validly cued ones, a probe question (“Prediction correct?”) was presented centrally, alongside two answer options (“Yes”/“No”) on the lower left/right side of the screen, which could be answered via button press with the right (i.e., ungloved) hand. If an answer was given within 1.5 s, feedback (“correct”/“wrong”) appeared afterwards for 0.75 s; otherwise, a visual reminder to answer more quickly was shown.

Each participant's recording session consisted of eight experimental blocks, with 32 trials each (balanced across task conditions within each block), resulting in 256 trials total. Depending on the duration of hand positioning, the delay period and occurrence of catch trials, each trial lasted between 8 and 15 s. On average, the main experimental session took about 60–65 min. To familiarize themselves with all conditions and cue meanings, participants performed a prior training session before the start of the recorded sessions until they felt confident to complete the task. Between experimental blocks, participants could rest as much as they needed.

### 2.4 Data Preprocessing and Analysis

Preprocessing was performed in MATLAB (MathWorks, version 2023b), with the *Edf2Mat* Matlab Toolbox (designed and developed by Adrian Etter and Marc Biedermann at the University of Zurich) used for the conversion of EyeLink 1000 edf files; all statistical analysis steps were performed via MATLAB and JASP (Version 0.95.4). To derive hand movement data, finger flexion was recorded from seven sensors (two from each finger, one excluded due to technical difficulties) on a data glove (5DT Data Glove 14 Ultra MRI, 60 Hz sampling rate, full‐speed USB 1.1 connection). Recorded data were averaged across all sensors and translated to a virtual hand position based on each participant's individual glove calibration (see above). For analysis of gaze‐hand tracking accuracy, hand movement data were interpolated to 1000 Hz to match the eye tracker's recording frequency.

Analysis of the pupillometry data followed common guidelines (Steinhauer et al. 2022). The continuous pupil size measurements were segmented to select two relevant time windows per trial, the delay period (0–3000 ms post cue appearance) and the execution period (0–2000 ms post virtual hand appearance). As our analysis focused on pupil size changes, all values were then re‐adjusted to a trial‐specific baseline, defined as the mean pupil size during the 200 ms before the respective time window. Via a recorded reference of defined size, pupil size measures were converted from arbitrary units to square millimeters.

Gaze‐ and hand position data were similarly segmented (with the execution time window also including the 500 ms before the instructed movement onset, that is, virtual hand appearance, to also include the ‘Ready’ signal), separated into *x*‐ (horizontal) and *y*‐ (vertical) direction, and re‐adjusted with the exact midpoint of the screen as the zero baseline. Gaze‐ and virtual hand position values were then converted from position on the screen to degrees of visual angle. For each trial, the gaze‐hand tracking accuracy time course was calculated as the offset between position of the visual target (i.e., the red dot on the tip of the virtual hand's middle finger) and gaze position. Overall accuracy per trial was calculated by the root mean square error (RMSE) of this offset. Specific hand‐ and eye movements during the execution phase were detected, defined as any time window where hand‐ or eye movement velocity exceeded a threshold of 20% of total hand movement range per second (or 5° visual angle per second, respectively). For each extracted movement, its onset, duration, and amplitude were calculated, as well as the number of saccades and mean saccade amplitude per trial.

Missing pupil size or gaze position data (e.g., due to blinks) was detected with the automatic algorithms of the EyeLink system. Data from trials with a continuous period of > 500 ms or more than 20% (pre‐registered: 40%) of the entire time window missing were automatically excluded from our analysis. In minor cases of missing data, values were substituted via cubic interpolation from data 50 ms before and after the missing section. In addition to this automated algorithm, all raw data were visualized and inspected manually to identify possible technical issues or artifacts not recognized otherwise, with affected trials corrected or discarded. Across participants, 99.0% of trials were found valid for the delay period (SD: 1.4%) and 98.6% for execution period (SD: 2.4%). Importantly, the valid trial rate of all participants was markedly above the threshold for exclusion defined in pre‐registration (70% valid trials), with the lowest valid rates at 95% and 87%, respectively. Therefore, no participant had to be excluded due to poor or missing data.

Previous studies have shown that eye movements systematically interfere with the measured pupil size, especially due to the pupil foreshortening error, that is, a foreshortening of the recorded pupil image as the eye rotates away from the camera (Brisson et al. 2013; Hayes and Petrov 2016). To account for this potential confound, we calculated the corrected pupil size based on the geometric model as described by Hayes and Petrov (2016). We used the distances between eye, camera, and data of subjects' gaze position on the screen to derive the cosine of the angle between the eye‐to‐camera and eye‐to‐gaze position axes. Measured pupil size values were divided by the square root of this cosine to determine the corrected pupil size. This corrected size was used for all further analysis, both during the execution phase, where participants were required to make eye movements and during the delay phase, where participants were instructed to fixate on the visual target, but systematic changes in gaze position might still have confounded our measurements.

For pupil size data during the delay phase, the stimulus‐locked mean time course and standard deviation were calculated on a single subject level for each condition, that is, combination of factors *predicted visuomotor mapping* (congruent, incongruent), and *movement type cueing* (cued, uncued), averaging across up to 64 trials each. As an overall measure of pupil size change within the delay phase, we also calculated the condition‐specific mean pupil size from 500 to 3000 ms post appearance of the visual cue for each participant, then used the results to perform a group‐level repeated‐measures analysis of variance (rmANOVA) with factors *visuomotor mapping* and *movement type cueing*. To determine the exact periods of significant differences between the (averaged) pupil size time series data of different conditions, we employed a nonparametric permutation cluster‐based analysis (Maris and Oostenveld 2007), implemented manually within our analysis. Specifically, for each comparison of two condition averages, we calculated a cluster‐based *t*‐test statistic between both (t‐threshold/z‐critical value for single time points at 1.96 for a two‐sided test) to derive its corresponding test statistic. This observed test statistic was compared to the test statistics of 10,000 permutations, calculated from the time series signal of randomly switched conditions within participants. The *p*‐value of the actually observed cluster was then determined as the proportion of random *t*‐test statistics with an equal or larger test statistic than the one we recorded.

As described in the pre‐registration, we wanted to determine the maximal pupil change during the delay phase. During analysis, we observed a period of initial pupil constriction. Therefore, we chose to calculate a ‘valley‐to‐peak’ amplitude between the time points with the lowest and highest pupil size within the delay phase for each condition and participant. We calculated two separate amplitudes: For the first, we subtracted minimum pupil size in the ‘valley’ time window (range: 500–1200 ms post cue appearance) from the maximum pupil size in the ‘early peak’ window (range: 800–2000 ms post cue appearance). In the second time window, minimum pupil size in the ‘valley’ time window was instead subtracted from the maximum pupil size in the ‘late peak’, window (range: 2500–3000 ms). For each amplitude, the associated duration, that is, distance between time points was also calculated, while ensuring that the peak time point always occurred after the valley time point. The results were analyzed via group level rmANOVAs with factors *visuomotor mapping* and *movement type cueing*.

Analysis of the pupil size during the execution phase was performed concordantly, by first calculating the stimulus‐locked mean time course and standard deviation for each condition. This included the nested factor *expectation violation* (matched, violated) besides factors *predicted visuomotor mapping* and *movement type cuein*g, averaging across 48 trials (expectations matched) or 16 trials (expectations violated) per condition. To determine overall differences between pupil dilation during movement execution, a three‐way rmANOVA based on these factors was calculated for the average pupil size during movement execution (time window: 500–2000 ms post virtual hand appearance). To verifying that hand movement types (hand opening or closing) did not significantly affect pupil size, we also calculated means separated by hand movement for additional analyses.

To test whether expectation violation trials evoked a “surprise response” characterized by greater pupil dilation, a permutation cluster‐based analysis (parameters see above) was calculated to determine time periods of significant differences between trials with matched versus violated expectations. To evaluate whether expectation violation‐evoked pupil size changes differed depending on mapping and cueing conditions, we calculated the pupillary “surprise response” by subtracting, for each combination of factors (CC, CI, UC, UI), the average time course of trials with matched violations from ones with expectation violation. The resulting group means were used in a cluster‐based permutation analysis (see above), contrasting cued and uncued movement type or congruent and incongruent mapping, respectively.

To evaluate how gaze‐hand tracking performance during movement execution differed depending on trial conditions, the average time course of tracking accuracy was calculated on a single subject level for each condition. Additionally, condition means were calculated for tracking accuracy root‐mean square error (RMSE), hand movement onset, gaze movement onset (both relative to start of execution phase and hand movement onset), number of saccades per trial, mean saccade amplitude, and mean gaze acceleration. For all of these measures, three‐way rmANOVAs with the factors described above were performed. A cluster‐based permutation analysis with the same parameters as described above was performed on the mean tracking accuracy time courses of trials with matched and violated expectations. Following up on significant main or interaction effects, we calculated post hoc *t*‐tests; these contrasts were Holm‐adjusted for multiple comparisons.

To test for a possible confounding effect of eye closure rate on pupil size and gaze position measures, we calculated the condition means of the number of blinks and the total blink duration during the delay and execution phase, respectively. These were then subjected to rmANOVAs with the factors described above. Similarly, we evaluated if fixation patterns during the delay phase differed between task conditions, possible confounding pupil size measurements. The mean offset between fixation target and gaze position in the *x*‐ (horizontal) and *y*‐ (vertical) direction was calculated as the RMSE from 0 to 3000 ms post cue appearance, then again subjected to rmANOVAs. Note however that the measures described above, that is, exclusion of trials and interpolation of values during blink periods and pupil size correction for the PFE should account for a possible confounding effect of these elements.

Finally, in a pre‐registered exploratory analysis, we tested if pupil dilation during the delay period correlated with tracking performance during movement execution. For each participant, a single trial‐based correlation analysis (Pearson correlation) was performed between pupil size change in the preparation phase (mean pupil size 500–3000 ms post cue appearance) and tracking performance, defined as either gaze‐hand‐tracking RMSE, onset of the first eye movement, or the number of saccades during movement execution. To test for significance on a group level, a one‐sample *t*‐test of the subjects' correlation coefficients was performed, with the significance level Bonferroni‐corrected for multiple comparisons.

To facilitate the evaluation of our pre‐registered hypotheses, we graphically indicate the statistical significance of any effects supporting one of the hypotheses in the respective figures (Figures 3, 4, 5). Asterisks signify a significance level of *p* < 0.05 (*), *p* < 0.01 (**), or *p* < 0.001 (***) respectively, and the relevant hypothesis is denoted.

## 3 Results

### 3.1 Task Compliance & Real Hand Movement

**FIGURE 2:**
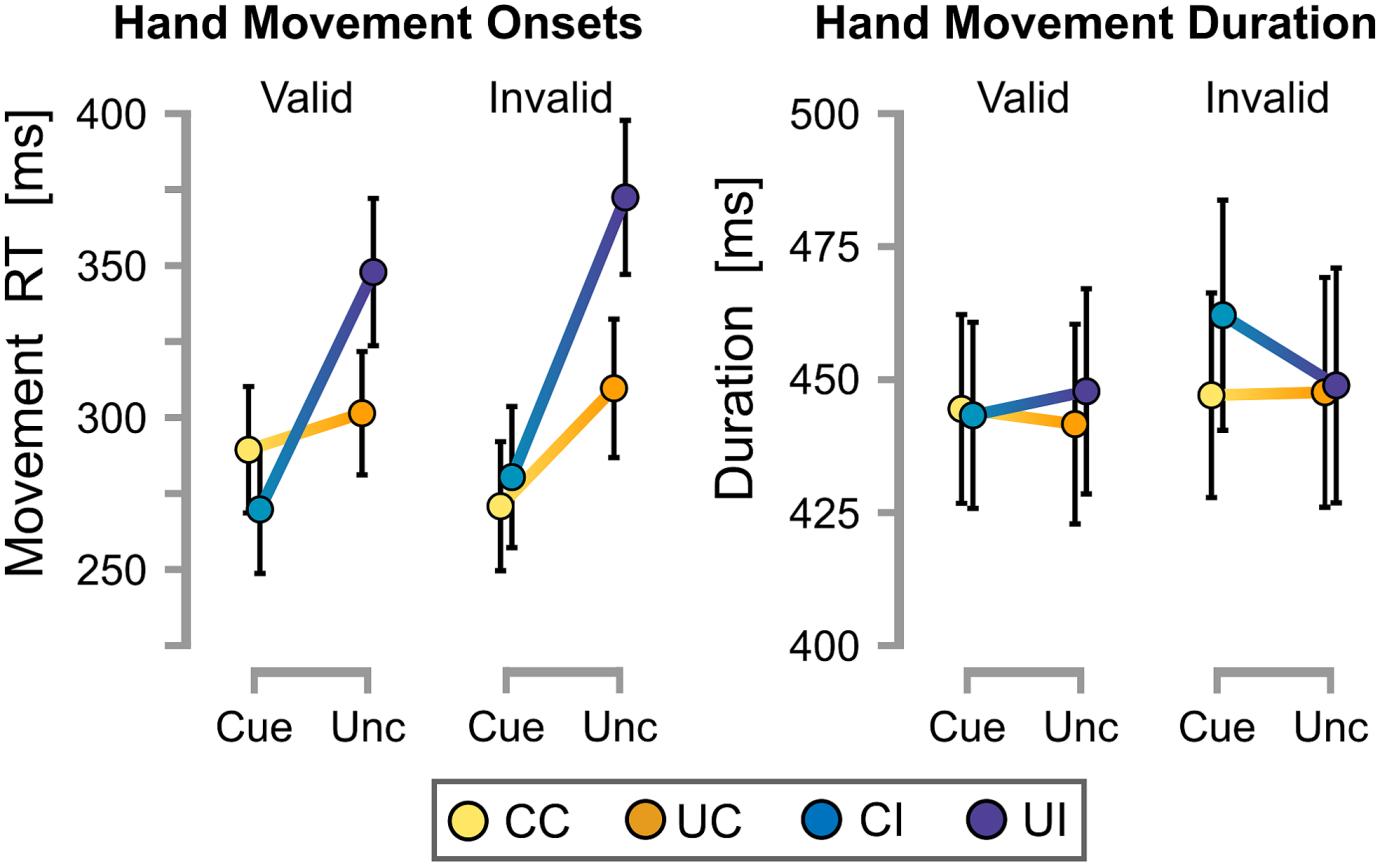
Hand movement execution. Mean hand movement onsets after the appearance of the virtual hand (left) and mean duration of the hand movements (right). Error bars represent standard errors of the mean.

Participants showed excellent task compliance, with, on average, 99.1% correctly executed hand movements (each participant > 94.1%) and 98.3% correct answers to the catch trials probing the cued visuomotor mapping (each participant > 89.8%). On average, hand movements were initiated 306 ± 132 ms after the appearance of the virtual hand model and lasted for 445 ± 110 ms (Figure 2). A three‐way rmANOVA revealed a significant effect of movement type cueing on movement onset (*F*_(1,38)_ = 33.809, *p* < 0.001, η^2^_p_ = 0.471), with pre‐cued movements initiated on average 55 ms earlier than uncued ones. There was also a significant effect of predicted visuomotor mapping (*F*_(1,38)_ = 16.123, *p* < 0.001, η^2^_p_ = 0.298), with participants initiating hand movements on average 25 ms later when expecting incongruent visual feedback. Additionally, there was a significant interaction effect of cueing and mapping (*F*_(1,38)_ = 23.499, *p* < 0.001, η^2^_p_ = 0.382), with movement onsets especially delayed for uncued trials where participants expected incongruent feedback. There was no significant effect of expectation violation on movement onsets, but a significant interaction of expectation violation and visuomotor mapping (*F*_(1,38)_ = 9.339, *p* = 0.004, η^2^_p_ = 0.197). Hand movement durations were comparable across conditions and factors (no significant main or interaction effect).

### 3.2 Pupil Size Changes Related to Motor Planning and Visuomotor Prediction

We hypothesized that during movement preparation, the cue‐induced pupil dilation should reflect the allocation of cognitive resources devoted to the generation of visuomotor predictions (incongruent > congruent, H1) and motor planning (cued > uncued, H2).

**FIGURE 3:**
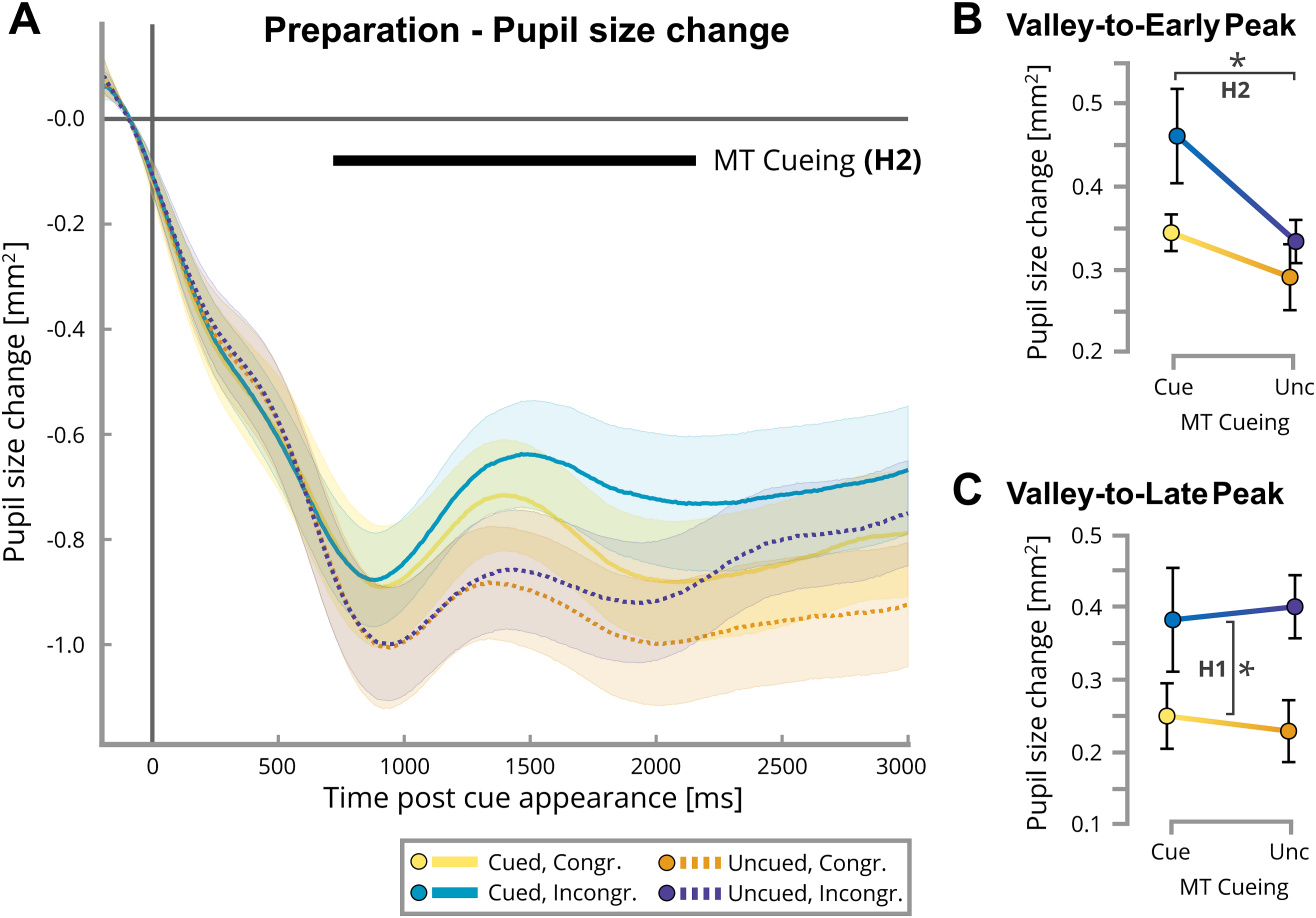
Preparation phase. (A) Pupil size change in the delay period during the first 3 s after cue appearance, relative to baseline (200 ms before cue appearance). Each line displays one condition, that is, combination of factors movement type (MT) cueing (cued, uncued) and predicted visuomotor mapping (congruent, incongruent), averaged across all subjects. Shaded areas indicate the between‐subject standard errors of the mean. The black bar indicates a cluster of time points showing a significant main effect of movement type cueing, with larger pupil dilation in cued > uncued trials (*p* < 0.05, determined via cluster‐based permutation tests with *n* = 10,000). (B, C) Group level means of the valley‐to‐peak pupil dilation analysis, that is, the pupil size difference between the point of lowest pupil size (‘valley’, time window: 500–1200 ms) and the point of highest pupil size in either (B) the ‘early peak’ time window (800–2000 ms) or (C) the ‘late peak’ time window (2500–3000 ms). Error bars represent standard errors of the mean. For the early peak (B), the main effect of *movement type cueing* was significant (*p* = 0.030), with larger pupil dilation when the movement type was cued > uncued, confirming H2; the other main and interaction effect were not significant. For the late peak (C), the main effect of *visuomotor mapping* was significant (*p* = 0.023), with larger pupil dilation when incongruent > congruent mappings were cued, confirming H1; the other main and interaction effect were not significant.

For the mean pupil size (500–3000 ms post cue appearance, see Table 1) a two‐way rmANOVA revealed a significant effect of *movement type cueing* (*F*_(1,38)_ = 6.641, *p* = 0.014, η^2^_p_ = 0.149). Accordingly, the cluster‐based permutation analysis of pupil size time courses (Figure 3A) revealed a period of significant (*p* = 0.019) difference between cued and uncued trials from 718 to 2158 ms post cue appearance, with increased pupil dilation when movement type was cued in advance compared to when it was left ambiguous. The main effect of *visuomotor mapping* and the interaction effect were not significant.

**TABLE 1:** Pupil size change during the delay phase, with standard errors of the mean.

Task conditions		Pupil size measures		
Mapping	Cueing	Mean size during delay phase	Valley‐to‐early peak amplitude	Valley‐to‐late peak amplitude
Congruent	Cued	−0.811 ± 0.105 mm^2^	0.345 ± 0.056 mm^2^	0.250 ± 0.098 mm^2^
	Uncued	−0.929 ± 0.106 mm^2^	0.291 ± 0.053 mm^2^	0.229 ± 0.092 mm^2^
Incongruent	Cued	−0.722 ± 0.098 mm^2^	0.461 ± 0.097 mm^2^	0.382 ± 0.126 mm^2^
	Uncued	−0.859 ± 0.100 mm^2^	0.334 ± 0.055 mm^2^	0.400 ± 0.087 mm^2^

Group level averages per condition (Figure 3A) showed a common pupil dilation peak in the middle of the delay period, as well as an increase in pupil size towards the end of the phase. To test if task conditions affected pupil dilation differently during these two time periods, we calculated two separate valley‐to‐peak amplitudes, that is, maximum pupil size changes (see Table 1; this separation was not pre‐registered). For the valley‐to‐early‐peak (Figure 3B), a two‐way rmANOVA confirmed a significant effect of movement type cueing (*F*_(1,38)_ = 5.066, *p* = 0.030, η^2^_p_ = 0.118), with a larger pupil dilation for cued > uncued trials, but no significant effect for predicted mapping nor an interaction. For the valley‐to‐late‐peak (Figure 3C), in contrast, there was a significant effect of visuomotor mapping (*F*_(1,38)_ = 5.575, *p* = 0.023, η^2^_p_ = 0.128), with larger pupil dilation for trials where incongruent > congruent mappings were predicted, but no significant effect for movement type cueing nor an interaction. Control analyses (Figure S1A) showed that eye blink behavior was comparable across conditions; fixation offset in the vertical direction slightly but significantly increased when movements were pre‐cued, though pupil size corrections should account for a possible related confound.

### 3.3 Pupil Size Changes During Task Execution

**FIGURE 4:**
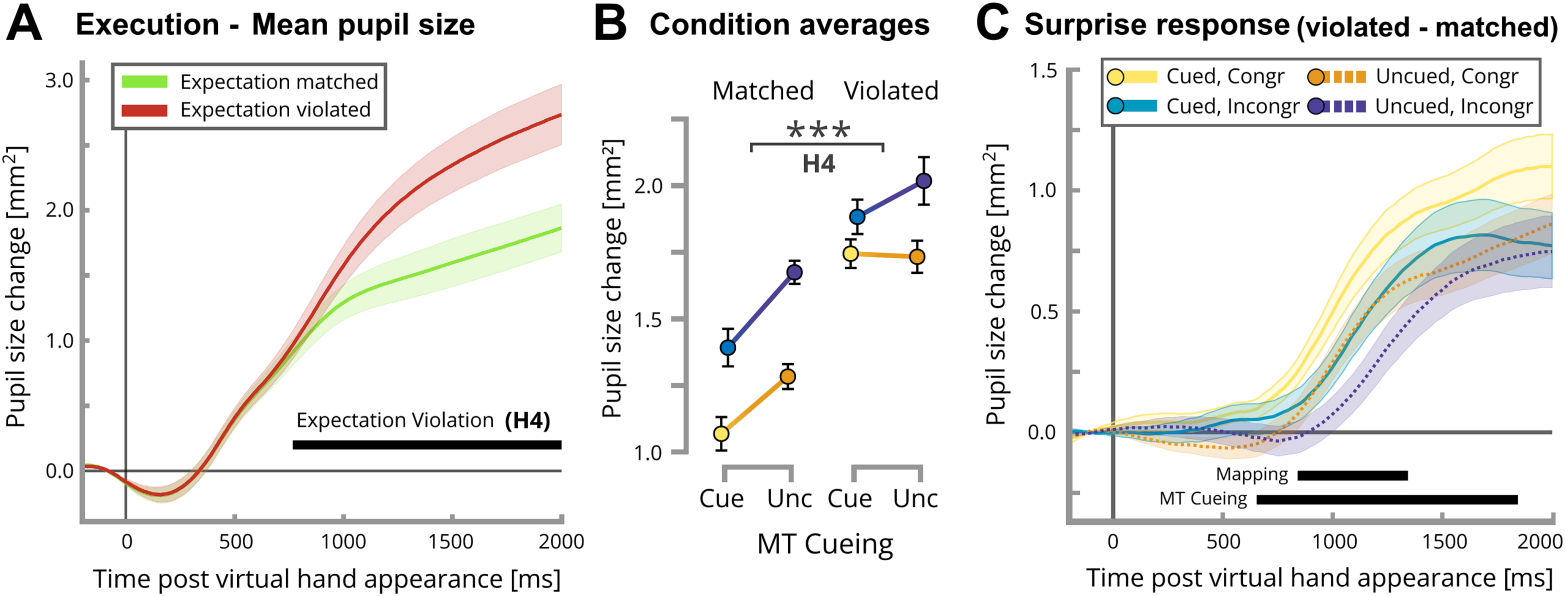
Pupil size during the execution phase. (A) Pupil size changes during the execution of hand movements with visual hand movement feedback matching the cued expectations (green line) versus violating them (red line). Each line displays the group level average relative to baseline; 0 marks the onset of the execution period with appearance of the virtual hand. Shaded areas indicate between‐subject standard errors of the mean. The black bar designates a significant cluster (*p* < 0.001, determined via cluster‐based permutation tests with *n* = 10,000) of time points with a significant main effect of expectation violation. Pupil dilation was larger in trials where visuomotor mapping expectations were violated > matched; confirming H4. (B) Group level mean pupil size during movement execution (500–2000 ms) relative to baseline; error bars indicate between‐subject standard errors of the mean. In addition to the significant main effect of expectation violation (*p* < 0.001, confirming H4), pupil dilation was significantly larger when incongruent > congruent mappings had been cued (*p* < 0.001), and when movement type had been left unspecified (uncued > cued, *p* = 0.001). Moreover, there was a significant interaction between expectation violation and movement type cueing (*p* = 0.012) and another between expectation violation and visuomotor mapping (*p* = 0.024); the other interaction effects were not significant. (C) Average pupillary “surprise responses” per condition; that is, the mean pupil size differences between trials were expectations were violated minus matched. The black bars designate clusters of time points with a significant (*p* < 0.05) main effect of predicted visuomotor mapping (*p* = 0.041) and movement type cueing (*p* = 0.012), respectively. See Results for details.

We then tested for the hypothesized pupillary “surprise response”, reflected by increased pupil dilation during movement trials in which the visual movement feedback violated the cued visuomotor expectations (*Hypothesis H4*). As expected, a rmANOVA of the group mean pupil size 500–2000 ms post virtual hand appearance showed a clear, significant main effect of expectation violation (*F*_(1,38)_ = 64.231, *p* < 0.001, η^2^_p_ = 0.628), with increased pupil dilation in response to violated > matched expectations (mean difference in pupil size = 0.49 mm^2^). A cluster‐based permutation analysis (Figure 4A) revealed that the expectation violation effect was significant between 768 and 2000 ms after the appearance of the virtual hand (*p* < 0.001), that is, largely after movement execution had finished.

We then calculated a three‐way rmANOVA of pupil size during movement execution to test for possible conditional effects on pupil dilation (Figure 4B). This revealed that cued visuomotor mapping had a significant effect on pupil diameter (*F*_(1,38)_ = 35.178, *p* < 0.001, η^2^_p_ = 0.481), with relatively larger pupil dilation when participants had expected incongruent compared with congruent mappings. There was also a significant interaction of cued visuomotor mapping and expectation violation (*F*_(1,38)_ = 5.496, *p* = 0.024, η^2^_p_ = 0.126); that is, in trials in which the seen movement violated, compared with matched cued expectations, pupil dilation was stronger when congruent compared with incongruent visuomotor mappings had been cued. Movement type cueing also had a significant main effect (*F*_(1,38)_ = 12.183, *p* = 0.001, η^2^_p_ = 0.243), with relatively smaller pupil dilation when movement type was cued in advance. Again, the interaction between movement type cueing and expectation violation was significant (*F*_(1,38)_ = 6.966, *p* = 0.012, η^2^_p_ = 0.155). Pupil dilation differences between trials with violated compared with matched expectations were modulated by cueing, demonstrating a stronger expectation violation effect (or “surprise response”) when to‐be‐performed hand movements were pre‐cued rather than uncued. The expectation violation effect was strongest in the CC condition (mean difference in pupil dilation between trials violating minus matching expectations = 0.68 mm^2^; all other conditions < 0.49 mm^2^), yet all individual expectation violation effects were significant (*p* < 0.004, corrected). All other interaction effects were non‐significant.

For a time‐resolved analysis of the above effects, we then calculated a cluster‐based permutation analysis (Figure 4C) on the condition time courses of the pupillary “surprise response”, that is, grand averages of trials with violated expectations minus matched expectations. This revealed a significant cluster (*p* = 0.012) for *movement type cueing* from 655 to 1843 ms after the virtual hand appeared, with a larger pupillary surprise response when movement type was cued > uncued. For *visuomotor mapping*, another cluster of significant difference (*p* = 0.041) was found from 841 to 1342 ms, with stronger pupillary surprise‐related dilation when a congruent > incongruent visuomotor mapping had been cued.

### 3.4 Gaze‐Hand Tracking

Next, we analyzed gaze‐hand tracking performance to test for the hypothesized effects of expectation violation, visuomotor congruence, and movement cueing on execution (*Hypotheses* *H4*–*H6*).

**FIGURE 5:**
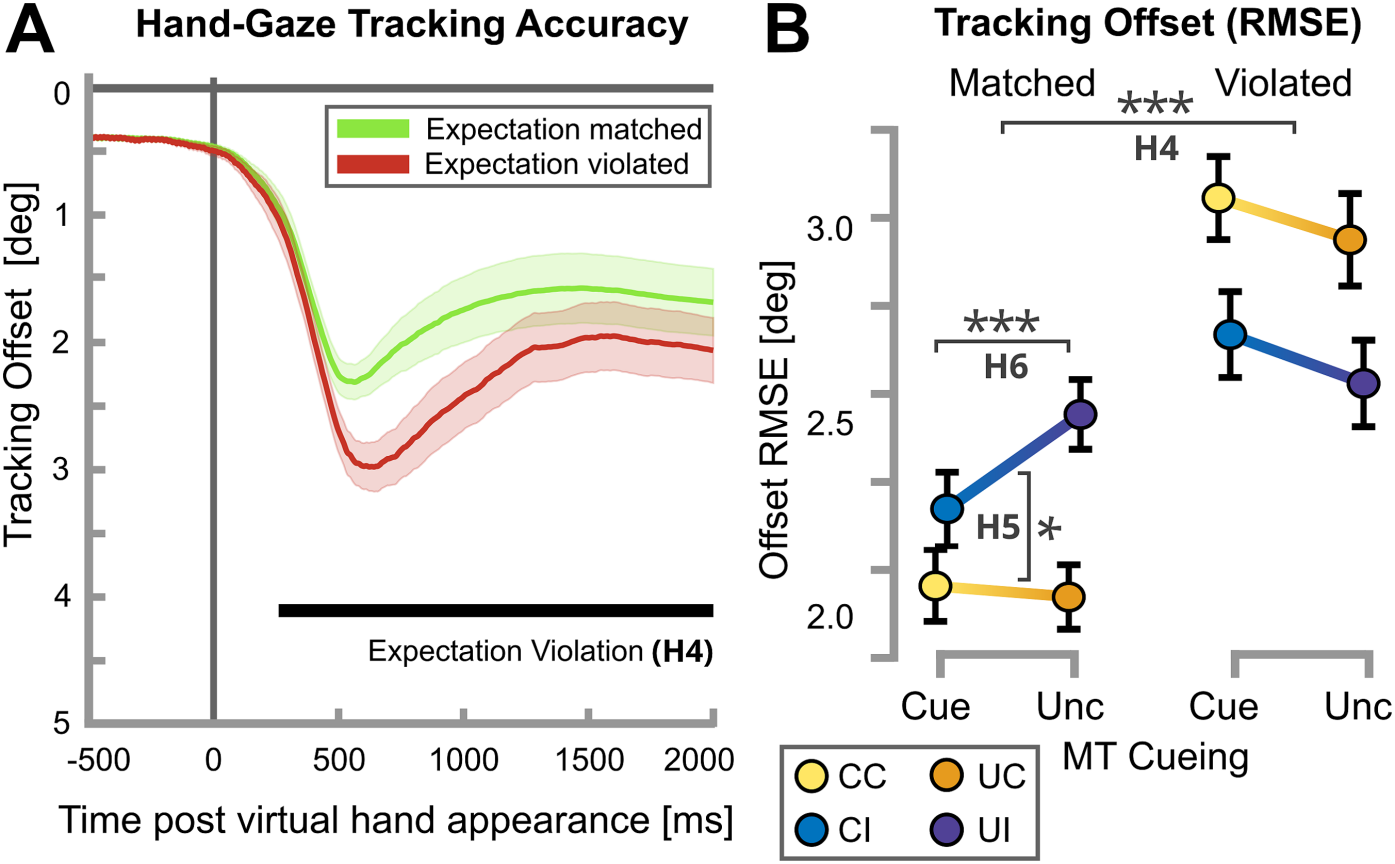
Gaze‐hand tracking performance. (A) Mean gaze‐hand tracking offset during movement execution; that is, the mismatch between the visual target's position (red dot at the tip of the virtual hand's middle finger) and the participants' gaze position on the screen. Shown are the mean offsets of trials where the hand (and target) moved in accordance with cued visuomotor expectations (matched, green line) versus where it moved contrary to them (i.e., violated expectations, red line). The colored areas indicate between‐subject. The black bar designates the cluster of time points with a significant main effect of expectation violation (*p* < 0.05, determined via cluster‐based permutation tests with *n* = 10,000), that is, gaze‐hand tracking suffered from expectation violation. (B) Group means of gaze‐hand tracking offset (root mean square error within the time window 500–2000 ms post appearance of the virtual hand, see Methods); error bars indicate between‐subject standard errors of the mean. Tracking offset was significantly larger when expectations were violated than when they were matched (*p* < 0.001); confirming H4. Moreover, this expectation violation effect interacted with predicted visuomotor mapping (*p* = 0.008), and with movement type cueing (*p* = 0.005). For trials where expectations were matched, tracking offset was smaller for congruent compared to incongruent mappings (*p* = 0.007), confirming H5, and smaller for cued compared to uncued trials (*p* < 0.001), confirming H6. See Results for details.

First, we tested for effects of expectation violation on gaze‐hand tracking via a three‐way rmANOVA. RSME (i.e., performance error) was significantly larger in trials where the visual movement feedback violated > matched the cued expectations (main effect, *F*_(1,38)_ = 29.522, *p* < 0.001, η^2^_p_ = 0.437; *Hypothesis H4*). A cluster‐based permutation analysis revealed that this performance difference was significant (*p* < 0.001) at 260–2000 ms after the appearance of the virtual hand (Figure 5A,B). Furthermore, there was a significant interaction of expectation violation with movement type cueing on average gaze‐hand‐tracking RMSE (*F*_(1,38)_ = 8.716, *p* = 0.005, η^2^_p_ = 0.187, Figure 5B); that is, expectation violation more strongly impoverished gaze‐hand tracking when movements were cued > uncued. There also was a significant interaction of expectation violation with cued visuomotor mapping (*F*_(1,38)_ = 7.768, *p* = 0.008, η^2^_p_ = 0.170); that is, expectation violation more strongly impoverished gaze‐hand tracking when congruent compared to incongruent mappings had been expected. Post hoc contrasts revealed that the expectation violation effect (i.e., poorer gaze‐hand tracking in trials violating than those matching cued visuomotor expectations) was significant when subjects expected congruent mappings, with the strongest difference in the cued, congruent condition (CC: *t*_(38)_ = 5.812, *p* < 0.001; UC: *t*_(38)_ = 5.250, *p* < 0.001). In contrast, there was no expectation violation effect on tracking under expected incongruent mappings (CI: *t*_(38)_ = 2.442, *p* = 0.232; UI: *t*_(38)_ = 0.457, *p* = 1). Furthermore, there was a statistical trend for a three‐way interaction among the above factors (*F*_(1,38)_ = 3.615, *p* = 0.065, η^2^_p_ = 0.087).

As expectation violation had a strong effect on gaze‐hand tracking, to further study the hypothesized effects of visuomotor mappings (H5) and movement type cueing (H6) on tracking performance (see Figure 5B), we then analyzed only trials with valid cueing, that is, matched expectations, in a separate two‐way ANOVA. Here, we found a significant effect of predicted visuomotor mapping (*F*_(1,38)_ = 8.071, *p* = 0.007, η^2^_p_ = 0.295), with better gaze‐hand tracking performance under congruent compared with incongruent visuomotor mappings (*t*_(38)_ = 2.841, *p* = 0.022; *Hypothesis H5*). We also found an effect of movement type cueing (*F*_(1,38)_ = 15.915, *p* < 0.001, η^2^_p_ = 0.295), with better performance when the movement type was pre‐cued (*t*_(38)_ = 3.989, *p* < 0.001; *Hypothesis H6*), and a significant interaction of cueing and mapping (*F*_(1,38)_ = 22.599, *p* < 0.001, η^2^_p_ = 0.373). Post hoc tests revealed that this effect was driven by a cueing advantage only for incongruent mappings: Here, when movement type was pre‐cued, the subsequent gaze‐hand tracking offset was significantly reduced compared to when it was left uncued (*t*_(38)_ = 5.418, *p* < 0.001). For congruent visuomotor mapping trials, in contrast, there was no such cueing advantage, with tracking offset in congruent visuomotor mapping trials similarly low for both cued and uncued trials (*t*_(38)_ = 0.865, *p* = 1). This difference in cueing advantage implied that, in cued trials, there was no performance difference between moving under congruent versus incongruent feedback (*t*_(38)_ = 1.608, *p* = 0.813).

The analysis of further eye movement kinematic parameters (saccade onset and amplitude, and tracking smoothness as indicated via gaze acceleration) overall supported these results; that is, partly showing corresponding effects: Again, we found decreased tracking performance (later saccade initiation, larger and more abrupt saccades) for expectation violation, better performance under validly cued standard mappings, and earlier saccade initiation when movements were pre‐cued (see Figure S2). Finally, pupil dilation during the delay phase did not significantly correlate with better tracking performance during movement execution.

## 4 Discussion

Here, we used a VR‐based, delayed‐movement task with concurrent eye tracking to examine the oculomotor correlates associated with planning movements under standard versus nonstandard visual hand movement feedback (i.e., updating of visuomotor expectations); and violation of said cued expectations during execution. Our key findings were: During preparation, pupil diameter increased when the movement was pre‐cued (versus left unspecified), and when nonstandard visual movement feedback was expected. Expectation violation impaired tracking performance and evoked a strong pupil dilation, which was strongest when participants had prepared specific (cued) movements and expected standard visuomotor mappings. Finally, gaze‐hand tracking performance decreased under nonstandard mappings, but significantly less so when the to‐be‐executed movement had been pre‐cued. We now discuss these findings in more detail.

Firstly, as predicted (H1), we observed larger pupil dilation when participants expected incongruent compared with congruent visual movement feedback. This difference was pronounced during the later parts of the pupillary cue response (around 1–2 s post cue appearance) and became significant at the late peak. In line with our hypotheses, one interpretation of this effect is that cueing incongruent (nonstandard) visual hand position feedback led to the updating of internal visuomotor associations; that is, the sensory predictions of the internal forward model. These updates would require increased computational resources and could consequently be associated with increased mental effort, which is known to evoke pupil dilation (see Introduction). This interpretation would also be supported by studies linking pupil dilation to error‐based motor learning (e.g., Yokoi and Weiler 2022). However, pupil dilation may also have been evoked by the expectation of sensory conflict and a more challenging eye‐hand tracking task. Higher recruitment of cognitive control reliably evokes increased pupil dilation (Ariel and Castel 2014; Rondeel et al. 2015; cf. Van der Wel and Van Steenbergen 2018), and cue‐evoked expectations of response conflict alone can evoke a larger preparatory pupil response (Unsworth et al. 2023). More generally, pupil dilation prior to task performance often reflects expectations about the upcoming task difficulty (Irons et al. 2017; O'Shea and Moran 2016; White and French 2017). While our current study does not allow to differentiate between these interpretations, they need not be exclusive and may well be related. In sum, we propose the delay‐period, visuomotor mapping‐related pupillary responses likely reflected the mental effort associated with increased demand for cognitive resources when anticipating visual hand movement feedback conflicting with life‐long visuomotor expectations; potentially, also related to updating visual forward predictions.

Secondly, also as predicted (H2), pupil dilation significantly increased after cues specifying the to‐be‐executed movements during preparation, compared with when the cues left the movement ambiguous until execution. We propose that this indicates the recruitment of cognitive resources by motor planning (Rosenbaum 1980). Note that we observed significantly faster hand movement initiation in cued compared to uncued conditions, demonstrating a reaction time advantage of pre‐cueing movements. This suggests that, in our task, participants prepared a specific movement if cued correspondingly, but abstained from movement planning in the uncued condition until the respective movement‐specifying cue appeared in the execution period. It has been argued that the presentation of multiple response options may also, depending on context, lead to parallel motor planning (Cisek and Kalaska 2005; Klaes et al. 2011; but see Dekleva et al. 2018). However, if participants would have planned both possible movements in parallel, one would have expected larger pupil dilation in the uncued condition, and less of a reaction time difference between cued and uncued conditions. Similarly, the pattern of our results suggests that participants did not perceive the uncued delay period as a state of increased behavioral (task‐related) uncertainty, which one would expect to increase pupil dilation (Becker et al. 2024; Brunyé and Gardony 2017; Fan et al. 2023; Preuschoff et al. 2011). In line with our interpretation, pupil dilation has previously been related to cues limiting the range of possible motor responses after instructed delays (Adam et al. 2014; Moresi et al. 2008). A similar effect was reported in two studies examining re‐orientation of spatial attention, where directional cues predicting target position evoked larger pupil dilation compared to non‐predictive cues (Dragone et al. 2018; Lasaponara et al. 2019). Not last, our findings match those obtained from our brain imaging study with a similar task design (Quirmbach and Limanowski 2024), in which cortical motor system activity and reaction times suggested that if movements were left ambiguous, planning was reduced or delayed until execution. In sum, the most likely explanation of the cue‐related pupil dilation is that it reflected the recruitment of cognitive resources by motor planning. While pupil dilation was most pronounced when specific movements were cued and visuomotor incongruence was expected, the anticipated interaction effect did not reach statistical significance (refuting H3). The timing difference between the significant main effects of movement type cueing and visuomotor mapping cueing on pupil dilation may indicate that motor planning and the preparation for novel visuomotor mappings constitute two sequential, potentially independent processes.

Thirdly, we observed significant “surprise responses” when, during execution, the visual movement feedback violated the cued expectations; that is, when the virtual hand moved unlike predicted. These responses manifested themselves as increased pupil dilation, in line with our hypothesis (H4). Importantly, besides the significant main effect of expectation violation across conditions, there were conditional differences in the pupillary surprise response as revealed by two significant interactions: Pupil dilation was significantly larger when the movement was pre‐specified (i.e., cued > uncued), and when participants expected standard (i.e., congruent, compared with incongruent) visual movement feedback. The strength of expectations has been observed to modulate surprise‐evoked pupil dilation (Kloosterman et al. 2015; Preuschoff et al. 2011; Reisenzein et al. 2019; Satterthwaite et al. 2007; Yokoi and Weiler 2022), and accordingly, a higher unexpectedness of stimuli produces a greater pupil dilation (e.g., Becker et al. 2024; Bianco et al. 2020; Preuschoff et al. 2011). In this light, our result suggests that when participants knew which movement they had to execute (cued > uncued), they formed stronger (more precise) sensory expectations. Similarly, predictions of “standard” (congruent) visual movement feedback were likely stronger (more precise), because those associations (and forward models) had been learned and used life‐long. Yet crucially, we observed a significant pupillary surprise response even when nonstandard (incongruent) visuomotor mappings had been cued (but movements were accompanied by standard visual feedback). Overall, this result shows that predictions of visual movement feedback can be adjusted prior to execution (i.e., in the absence of movement and related sensory inputs), supporting the idea that forward models can be updated during motor planning, not only by sensory prediction errors (cf. Kuang et al. 2016).

Expectation violation also affected gaze‐hand tracking; that is, as predicted (*Hypothesis H4*), tracking was overall poorer when the visual movement feedback violated the cued expectations. Indeed, the effect of expectation violation was strong enough that when participants had to perform tracking under standard mappings, their performance was significantly worse when they had expected nonstandard mappings than when they had expected standard mappings. However, when expecting incongruent mappings, participants' subsequent performance did not differ significantly between incongruent or congruent visual feedback trials. This could mean that any anticipatory processes during the preparation for movements under expected visuomotor incongruence (indicated by significantly stronger pupillary responses during the delay, see above) did not directly influence eye‐hand coordination during subsequent movement execution. Alternatively, it could mean that even when unexpected, standard (congruent) visual hand movements were comparably easier to gaze‐track than nonstandard ones, resulting in a relatively smaller error during those unpredicted (surprise) trials. This needs to be answered by future work. Nevertheless, surprise responses as quantified by pupil dilation and gaze‐hand tracking showed, in principle, a consistent picture: In line with the surprise effect assessed via pupil dilation, the strongest detrimental expectation violation effect on tracking performance was observed in the cued, congruent condition. Furthermore, in the uncued, incongruent condition, there was virtually no expectation effect on tracking, and only a comparatively small expectation violation effect on pupil size.

Furthermore, gaze‐hand tracking performance in valid trials was overall poorer under nonstandard mappings; which confirmed our *Hypothesis H5* and mirrored previous eye‐hand coordination findings (Chen et al. 2016a; Dalecki et al. 2019; Hosseini et al. 2017; Landelle et al. 2016; Yeomans, Phillips, et al. 2021). Crucially, when the movement type was pre‐cued, this difference was significantly reduced, and not significantly different from tracking performance under standard visuomotor mappings. This suggests that, during motor planning, forward predictions of visual movement feedback could be successfully updated—benefiting subsequent eye‐hand coordination under nonstandard visuomotor mappings (cf. Chen et al. 2016b; Danion and Flanagan 2018; Landelle et al. 2016; Matsumiya 2021; Vercher et al. 1995; Yeomans, Phillips, et al. 2021). No significant cueing‐evoked difference was found for tracking under congruent feedback, which could suggest that for a standard mapping (i.e., following life‐long learned associations), motor planning may have been easier or occurred faster, so that pre‐cueing provided no behavioral benefit. Thus, while a cueing benefit on eye‐hand coordination was not observed under congruent (standard) visuomotor mappings, the significant cueing benefit under nonstandard mappings partly supports our *Hypothesis H6*.

Our results should be interpreted in light of the following limitations. Unexpectedly, participants exhibited pupil constriction during the first 900 ms of the delay period, which could have been caused by changes in luminance and/or residual cognitive processing during pre‐positioning of the hand based on visual feedback before each trial. Furthermore, we cannot conclusively determine to what extent eye movements in our task actually represented intended hand tracking or simply the parallel planning and execution of hand and (anti‐)saccade movements. Further research using prolonged and more complex hand movements could clarify this distinction.

In conclusion, our results suggest that oculomotor responses reflect processes related to motor planning and flexible forward prediction of sensory action consequences ahead of execution, and that specific expectations about visuomotor (in)congruency affect eye‐hand coordination and pupillary responses during subsequent execution.

## Author Contributions

**Jakub Limanowski:** conceptualization, methodology, validation, supervision, funding acquisition, resources, writing – review and editing, writing – original draft, project administration. **Felix Quirmbach:** writing – original draft, writing – review and editing, investigation, conceptualization, methodology, software, formal analysis, visualization, data curation, validation. **Sebastian Pannasch:** supervision, resources, writing – review and editing, validation, funding acquisition. **Annika Dix:** methodology, software, supervision, writing – review and editing, resources, validation. **Jens R. Helmert:** methodology, writing – review and editing, validation, resources.

## Funding

This work was funded by the German Research Foundation (DFG, Deutsche Forschungsgemeinschaft) as part of Germany's Excellence Strategy—EXC 2050/2—Project ID 390696704—Cluster of Excellence “Centre for Tactile Internet with Human‐in‐the‐Loop” (CeTI) of TUD Dresden University of Technology. J.L. was supported by a Freigeist Fellowship of the VolkswagenStiftung (AZ 97‐932).

## Conflicts of Interest

The authors declare no conflicts of interest.

## Supporting information


**Figure S1:** Eye closure and fixation accuracy patterns.
**Figure S2:** Secondary gaze‐hand tracking performance indicators.

## Data Availability

The behavioral and eye tracking data will be made available upon request.
