# Supplementary material for: Eye Tracking Insights Into Movement Preparation and Execution Under Nonstandard Visual Movement Feedback
Source: Psychophysiology. 2026 Aug 2;63(8):e70373. doi: 10.1111/psyp.70373 (PMC13430085; doi:10.1111/psyp.70373)
Supplement: Supplementary file 1 — Figure S1: Eye closure and fixation accuracy patterns. Figure S2: Secondary gaze‐hand tracking performance indicators. [file PSYP-63-e70373-s001.docx]

**Supplementary Material**

**Eye tracking insights into movement preparation and execution under nonstandard visual movement feedback**

**Authors:** Felix Quirmbach, Jens R. Helmert, Sebastian Pannasch, Annika Dix*, Jakub Limanowski*

*Annika Dix and Jakub Limanowski contributed equally.

**
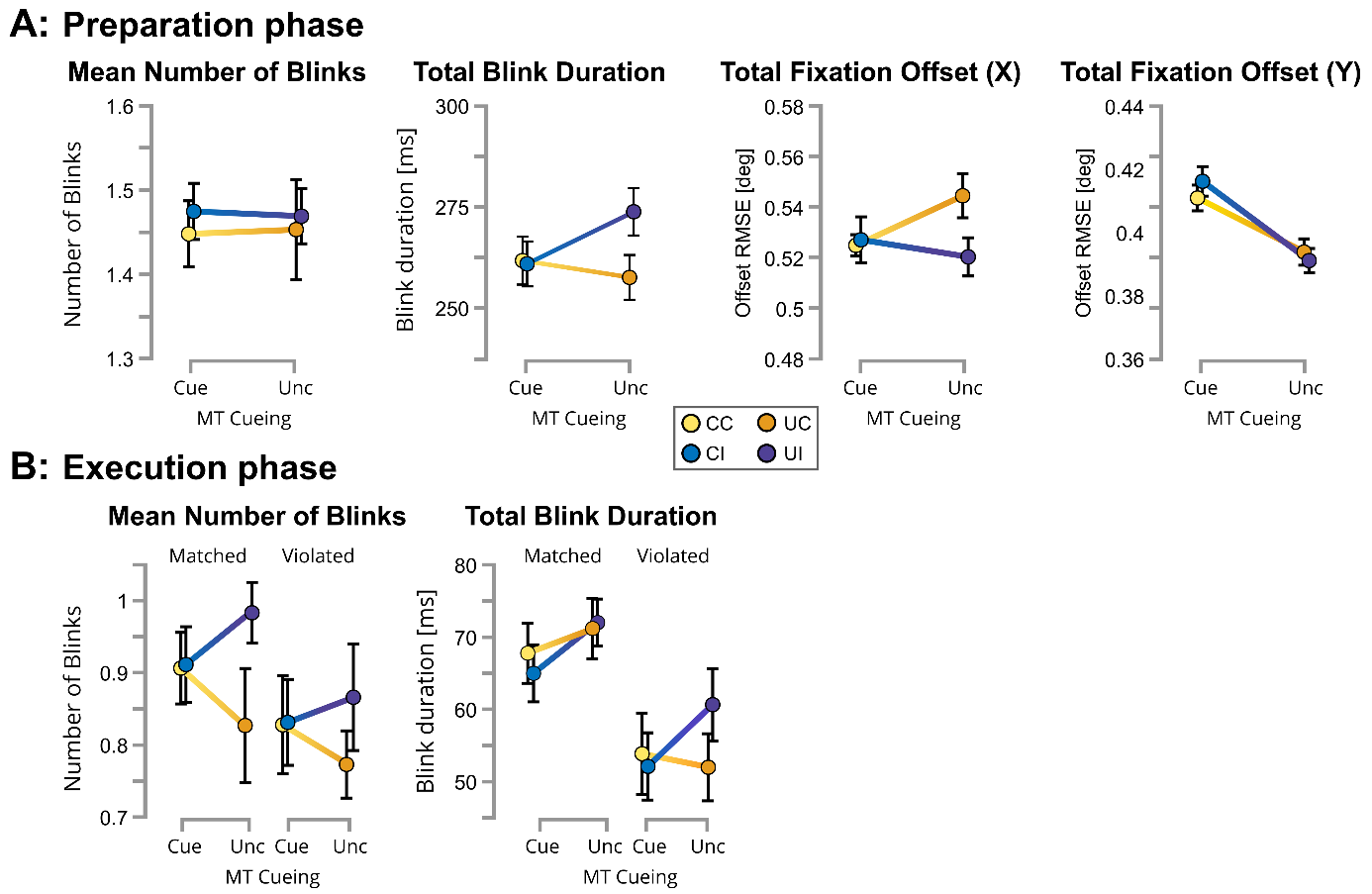
Fig. S1: Eye closure and fixation accuracy patterns**

Group means of control measures of participants’ gaze behavior, i.e. average number and total duration of eye closures (‘blinks’) during the preparation phase (A) and execution phase (B), and total gaze-fixation target offset in x- and y-dimension during the preparation phase (0 to 3000 ms post cue appearance). For the preparation phase, analysis via two-way rmANOVA revealed no significant effect of factors movement type cueing or expected visuomotor mapping on either the mean number or total duration of blinks. For fixation offset as quantified by the total offset RMSE, we found a significant interaction between cueing and mapping for the horizontal (x) dimension (*F*_(1,38)_ = 4.009, *p* = 0.052, η²_p_ = 0.095), and a significant main effect of cueing for the vertical (y) dimension (*F*_(1,38)_ = 17.771, *p* < 0.001, η²_p_ = 0.319), with higher offset if a specific movement type was pre-cued. For the execution phase, a three-way rmANOVA, with additional factor expectation violation revealed a significant interaction of cueing and mapping on the mean number of blinks (*F*_(1,38)_ = 5.123, *p* = 0.029, η²_p_ = 0.119) ), with more blinks for incongruent > congruent mapping only when movement type was not pre-cued, and for total blink duration a main effect of expectation violation (*F*_(1,38)_ = 9.921, *p* = 0.003, η²_p_ = 0.207), with overall shorter eye closures when expectations were violated rather than matched.

**Fig. S2: Secondary gaze-hand tracking performance indicators**

**
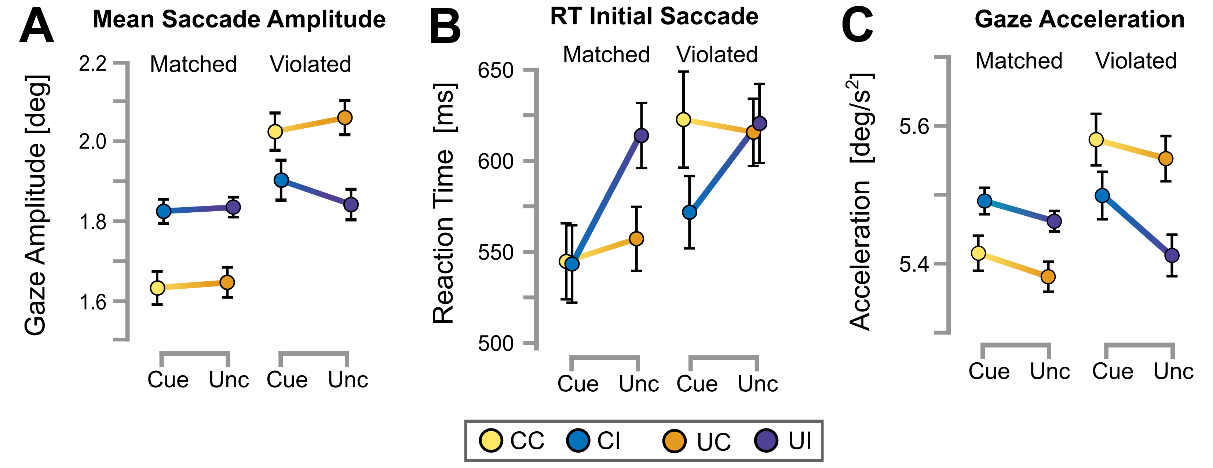
**

Group means of secondary indicators of gaze-hand tracking performance, i.e., (A) saccade amplitude during movement execution, (B) reaction time until initiation of the first tracking saccade, and (C) gaze acceleration during the execution phase; error bars indicate between-subject standard error of the mean. Analysis via three-way rmANOVAs revealed a significant effect of expectation violation for all measures, with violated expectations associated with increased saccade amplitude (*F*_(1,38)_ = 24.964, *p* < 0.001,
η²_p_ = 0.396), later saccade initiation (*F*_(1,38)_ = 20.666, *p* < 0.001, η²_p_ = 0.352), and higher gaze acceleration, i.e. more abrupt eye movements (*F*_(1,38)_ = 7.918, *p* = 0.008, η²_p_ = 0.172). Expectation violation furthermore interacted with predicted visuomotor mapping (*p* < .001, for all measures), as the performance decrease following violated expectations was especially pronounced when participants expected standard visuomotor mappings. For saccade initiation (B), there was also a significant main effect of movement type cueing (*F*_(1,38)_ = 12.222, *p* = 0.001, η²_p_ = 0.243), with earlier initiation when movements were pre-cued, and an interaction of cueing with predicted visuomotor mapping (*F*_(1,38)_ = 18.761, *p* < .001, η²_p_ = 0.331), as this cueing advantage was strong in trials under incongruent mapping, but not significant for congruent mappings. We also found a (non-significant) trend for an interaction of cueing and expectation violation (*F* = 3.347, *p* = 0.075, η²_p_ = 0.081). For gaze acceleration (C), there was also a significant effect for cueing (*F* = 5.002, *p* = 0.031, η²_p_ = 0.116), with higher acceleration (i.e., more abrupt saccades) when hand movements were pre-cued.
